# Patients as partners in a research advisory council role: describing the APERSU Patient Engagement Network

**DOI:** 10.1186/s41687-026-01001-8

**Published:** 2026-01-31

**Authors:** Sadia Ahmed, Marcia Bruce, D’Arcy Duquette, Veronika Kiryanova, Meron Seyoum, Simron Sidhu, Gloria Wilkinson, Fatima Al Sayah, Jeffrey A. Johnson, Arto Ohinmaa, Allison Soprovich

**Affiliations:** Alberta PROMs and EQ-5D Research and Support Unit (APERSU), School of Public Health, University of Alberta, Edmonton, Canada

**Keywords:** Patient and public involvement and engagement (PPIE), Patient-reported outcomes, Health outcomes research

## Abstract

Patient and public involvement and engagement (PPIE) is crucial for developing patient-centered healthcare research and improving health outcomes. While patient-reported outcome measures (PROMs) capture patients’ perspectives, meaningfully engaging patients in PROMs initiatives remains a challenge. This short report details the establishment and evolution of the Alberta PROMs and EQ-5D Research and Support Unit Patient Engagement Network (APERSU-PEN), a unique model designed to embed patient expertise and share practices throughout patient communities. Unlike many patient engagement groups focused on specific conditions, APERSU-PEN broadly integrates patient perspectives across the healthcare system. The network intentionally recruited experienced patient partners with advocacy and research backgrounds from targeted health and patient organizations. This approach enabled diverse representation and facilitated broad dissemination of PROMs awareness to various patient communities. This broad integration has most recently supported PROMs use in primary care. Key activities include the co-development of patient-friendly PROMs educational materials and active contributions sharing PROMs stories and experiences at various forums, supporting patient understanding and aiming to improve completion rates. The paper highlights challenges and their solutions, such as the broad scope of APERSU’s work, which was addressed by building this network. A critical success factor has been dedicated funding for the involvement of patients as partners, ensuring sustainable engagement and participation in external events. Flexible meeting options and cloud-based document sharing also accommodate busy schedules and geographical dispersion. APERSU-PEN exemplifies a transformative shift in healthcare culture and practice, where patients are recognized as “true end-users” of PROMs. This environment of mutual learning and respect between patients and researchers enhances the relevance and impact of PROMs. The model offers transferable guidance for other initiatives seeking to integrate PPIE meaningfully, ultimately increasing awareness of PROMs throughout patient communities. Future directions include evaluating member experiences and expanding diversity within the network to further strengthen patient engagement in PROMs and APERSU’s support services.

## Introduction

Patient and public involvement (PPIE) is essential for advancing health research that impacts patient-centered care. The Canadian Institutes of Health Research Strategy for Patient-Oriented Research calls for patients to be more involved in health research to improve health outcomes and enhance the healthcare systems [1]. Likewise, the UK's National Institute for Health and Care Research highlights that PPIE in research increases the quality and relevance and impact of research findings [2]. Effective partnerships between patients and researchers can be built on the principles of inclusiveness, goal and role clarity, training and capacity building, shared decision making, and strong, supportive leadership [3]. Over the last two decades, the customary view of patients as passive research subjects has given way to a model that recognizes them as active collaborators. Academia and accompanying policy directives now emphasize the incorporation of patients’ experiential knowledge into the entire investigative cycle. Terms such as “person with lived experience (PWLE), “patient partners” and “patient advocates” have developed to encompass various types of involvement [4, 5]. Here, we use the term “patient partners” to refer to patients, caregivers, and others who share insights from living with illness, using the healthcare system, and connecting with their communities. This evolution parallels broader moves toward patient‑centered care and the widespread adoption of patient‑reported outcome measures (PROMs) for both research and routine outcome measurement, which incorporates the patients’ voice in the assessment of health and healthcare quality [6].

This movement to PPIE has been gradual rather than abrupt. Throughout much of the twentieth century, research agendas were shaped almost exclusively by funders, investigators and clinicians, leaving patients with minimal agency. High‑profile advocacy movements, particularly within chronic‑disease communities, challenged that status quo. These groups demanded accountability, transparency and a decisive role for those living with the conditions under study. Advisory councils, community‑based participatory boards and national task forces have since institutionalized patient partnership, while models such as the International Association for Public Participation (IAP2) spectrum now articulate graded levels of involvement from consultation to empowerment [7]. Despite these advances, most engagement structures historically emerged within single-condition communities, leaving fewer established models for engagement that span the breadth of health conditions and services.

Amid growing momentum for patient engagement, increasing awareness of PROMs among patients and their communities is essential [8]. PROMs are designed to capture subjective dimensions such as pain, fatigue and psychosocial well‑being that traditional clinician‑reported metrics may overlook. While these tools align with patient-centered care, many PROMs initiatives challenge to engage the very individual whose experiences they aim to reflect in a meaningful and sustained way. PPIE is crucial for ensuring that PROMs are relevant, useful and effectively applied in both research and routine care, bridging the gap between measurement and meaningful impact on patient care.

Ongoing patient engagement in PROMs ensures sensitivity, accessibility, and contextual relevance [8]. These pose more importance when PROMs are implemented across conditions with varying symptom profiles or in geographically dispersed communities with diverse cultural and linguistic backgrounds. Patients who understand the purpose and methods behind PROMs can serve as invaluable partners in making these tools more relevant, inclusive and accessible [9]. However, this can only happen with dedicated support, formal structures and resources that promote awareness and empower patients to participate broadly in PROMs development and use [3]. Yet a persistent gap remains. PROMs initiatives that operate across the health system rarely have an accompanying engagement model capable of representing a wide spectrum of patient experiences. As PROMs implementation evolves, new models are needed to connect multiple patient groups, build shared understanding of PROMs and ensure that patients are engaged.

The Alberta PROMs and EQ‑5D Research and Support Unit (APERSU) exemplifies these dynamics, championing use of PROMs in both research and routine clinical practice and policy in the province of Alberta, Canada [6]. Because APERSU’s mandate spans the health system, it required an engagement strategy capable of bringing together diverse patient voices in a coordinated way. This prompted the development of the APERSU Patient Engagement Network (APERSU-PEN), conceived as a “network of networks” that connects advocates, patient leaders and community organizations whose experiences collectively reflect the broad scope of PROMs use in Alberta. The objective of this paper is to describe the development, implementation, and impact of the APERSU-PEN as a model for meaningful patient engagement in PROMs research and implementation practices.

## The evolution of the APERSU-PEN

APERSU, established in 2015, is a collaborative initiative between the government, health system, and academia for the implementation and use of PROMs. It is an example of embedded support services to facilitate information gathering, research activities and implementation strategies, incorporating the patients’ perspective. To date, we have issued more than 200 licenses to use the EQ-5D and support more than 300 end-users of PROMs in the province, across different health settings and populations. Although patient-focused, engaging patients as partners in a broad sense was challenging, as PROMs implementation, data use and reporting spans across conditions and populations. As the role of patient partners evolved and became well established in the province, our focus on patient engagement evolved and developed into the APERSU-PEN.

The initial purpose of the APERSU-PEN was to build a patient-facing engagement strategy, increase awareness of PROMs, and integrate patient perspectives into APERSU’s research and support services. Since its inception 18 months ago, the APERSU-PEN has evolved quickly into an active and motivated network, committed to advancing PROMs within the patient community and advocating for greater patient involvement in PROMs-related research, integration in routine care, completion rates, and healthcare decision-making. Table 1 illustrates the timeline of various knowledge mobilization activities the APERSU-PEN has participated in to spread and increase awareness of PROMs (Table 1).

**Table 1:** Timeline of APERSU-PEN knowledge mobilization PROMs activities

Activity	Month/Year	Outcome
Invited members to join APERSU-PEN	January 2024	Group established
Terms of reference finalized	March 2024	Commitment declared
Tip Sheet finalized	September 2024	Product co-created to increase PROMs awareness
Attendance at the APERSU-End-User meeting, panel presentation, town hall participation	October 2024	First patients to be involved, attend and actively contribute to the APERSU End-User meeting through presentations and panel participation
Annual check-in individual meeting	December 2024	APERSU-PEN members given the opportunity to share feedback individually and assess contributions
Attendance and workshop presentation at Primary Care Strategic Forum	May 2025	Co-developed workshop materials with administrators, shared patient experiences using PROMs, increased stakeholder engagement
Attendance, poster, workshop and oral presentation at AbSPORU Northwest Collaborative Forum	May 2025	Co-developed poster, workshop and oral presentation, shared patient stories of PROMs use, increased engagement
Tip Sheet translated to French	May 2025	Collaborated with Université de Sherbrooke (Marie-Eve Poitras’ lab [10]) to translate Tip Sheet
Planned: Evaluation using PEIRS-22	Fall 2025	Feedback to inform future planning, guide improvements and evaluate member satisfaction

**Fig. 1:**
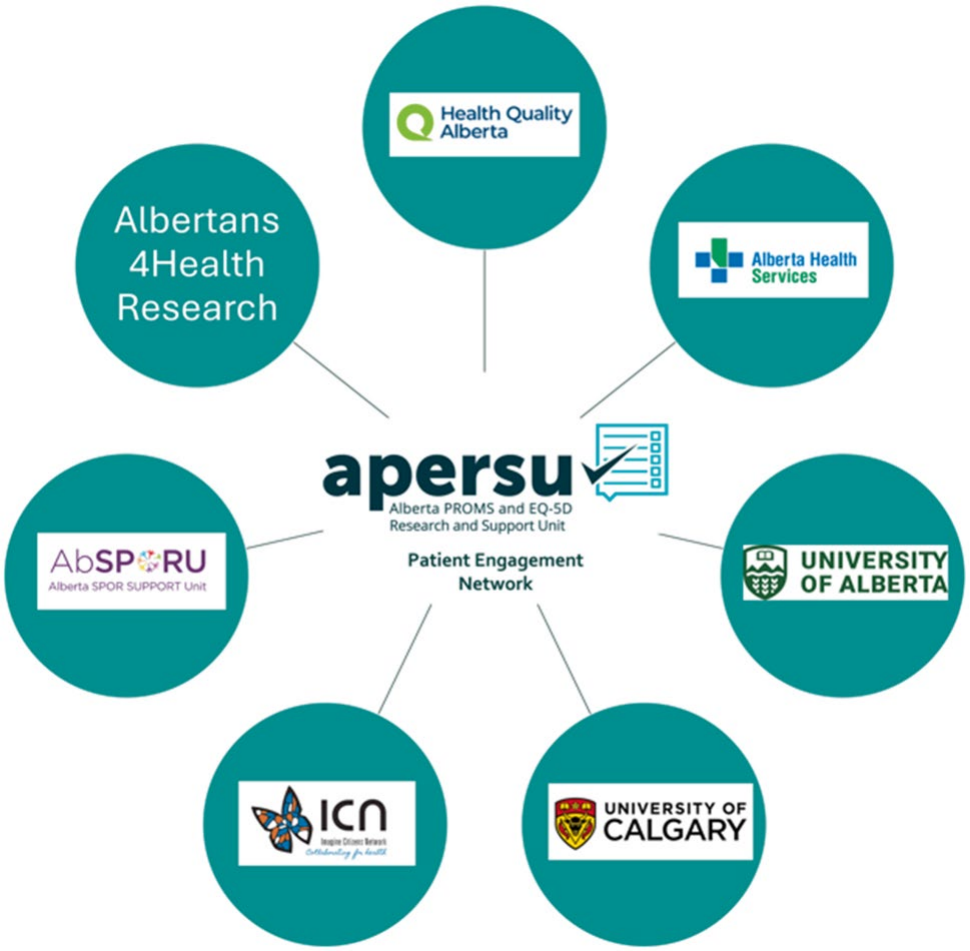
Alberta-based organizations represented by APERSU-PEN members

A unique feature of the APERSU-PEN model is its broad scope, allowing patient expertise to inform PROMs use across many different clinical environments. This has been especially beneficial to the primary care environment, where patients and their conditions and contexts are extremely diverse. Our work has already led to tangible results and impacts, including a growing network of patient advocates, increasing awareness and knowledge of PROMs within their communities, as Fig. 1 represents. Through our educational materials and outreach, as well as improved understanding of patient perspectives, our network members are influencing how PROMs are viewed by patients, clinicians and researchers. By creating patient-driven educational materials, participating in academic and healthcare events and sharing lived experiences, the APERSU-PEN is actively shaping how PROMs are understood, communicated and applied. We estimate that to date, the Tip Sheet has been given to over 200 people, and APERSU-PEN members have spoken to and presented to over 300 people in person at the events listed in Table 1. The network is an example of patient-researcher collaboration in action using authentic partnerships, co-developing innovative knowledge translation tools and driving long-term organizational impacts.

## Our approach

The APERSU end-user support team conducted an initial scoping review of patient engagement opportunities and practices throughout Alberta. We aimed to understand patient engagement theories and frameworks [1, 7, 11], to better refine our purpose and intention prior to recruitment. The Alberta SPOR Support Unit (AbSPORU) [9] was a critical partner in this step, providing guidance and support throughout the process. AbSPORU also provided seed grant support for initial engagement activities.

We intentionally recruited experienced, well-connected patients to be involved from targeted health organizations and purposeful contacts in the Alberta healthcare system to ensure the members were well-connected in existing patient communities; as shown with seven spokes connected to APERSU in Fig. 1. We started recruitment by connecting with the APERSU Board of Directors and the overarching health agencies in Alberta (Alberta Health, Alberta Health Services, Health Quality Alberta) for connections to targeted patient organizations. In addition, we advertised the opportunity with the AbSPORU Albertans4HealthResearch platform [12]. We were seeking long-term patient partners, with previous training and experience in advocacy and research practices, which were necessary to achieve the aim of spreading information and awareness of PROMs among patient communities. Fourteen potential patient partners indicated their interest by email, ten invitations were given to join the group, and nine attended the first virtual meeting (as a meet and greet). The patient members of the APERSU-PEN bring firsthand experiences with managing acute and chronic conditions across diverse healthcare settings. As many patient advocacy groups are defined by a specific condition, this was a unique opportunity to connect across the broader patient community. The need for individual and organizational diversity was recognized considering the broad scope of this work. Partners often draw on their networks to gather feedback from the broader patient and caregiver community, ensuring that a wider range of voices is considered [12]. The APERSU-PEN has diverse representation in terms of age, sex, education, healthcare utilization, health conditions, cultural practices and abilities. Although it was not a requirement, the initial members of the APERSU-PEN all had Patient and Community Engagement (PaCER) certifications [13]. PaCER is a University of Calgary Continuing Education program that teaches patients and community members how to meaningfully engage in health research to influence healthcare planning, practice, and policy [13].

The terms of reference were co-created over two meetings. Group dynamics were strong from the outset, as many members were already familiar with one another through PaCER and other previous collaborations, eliminating the need for formal team-building activities. Subsequent meetings hosted guest speakers from APERSU and other collaborators to provide examples and foster deeper understanding of PROMs use in Alberta. Ongoing opportunities for capacity building are also offered. We have an extensive collaborative approach, offering flexibility over the spectrum of engagement [7, 11] and making decisions based on full group consensus, not only those in attendance.

## Activities to date

**Fig. 2:**
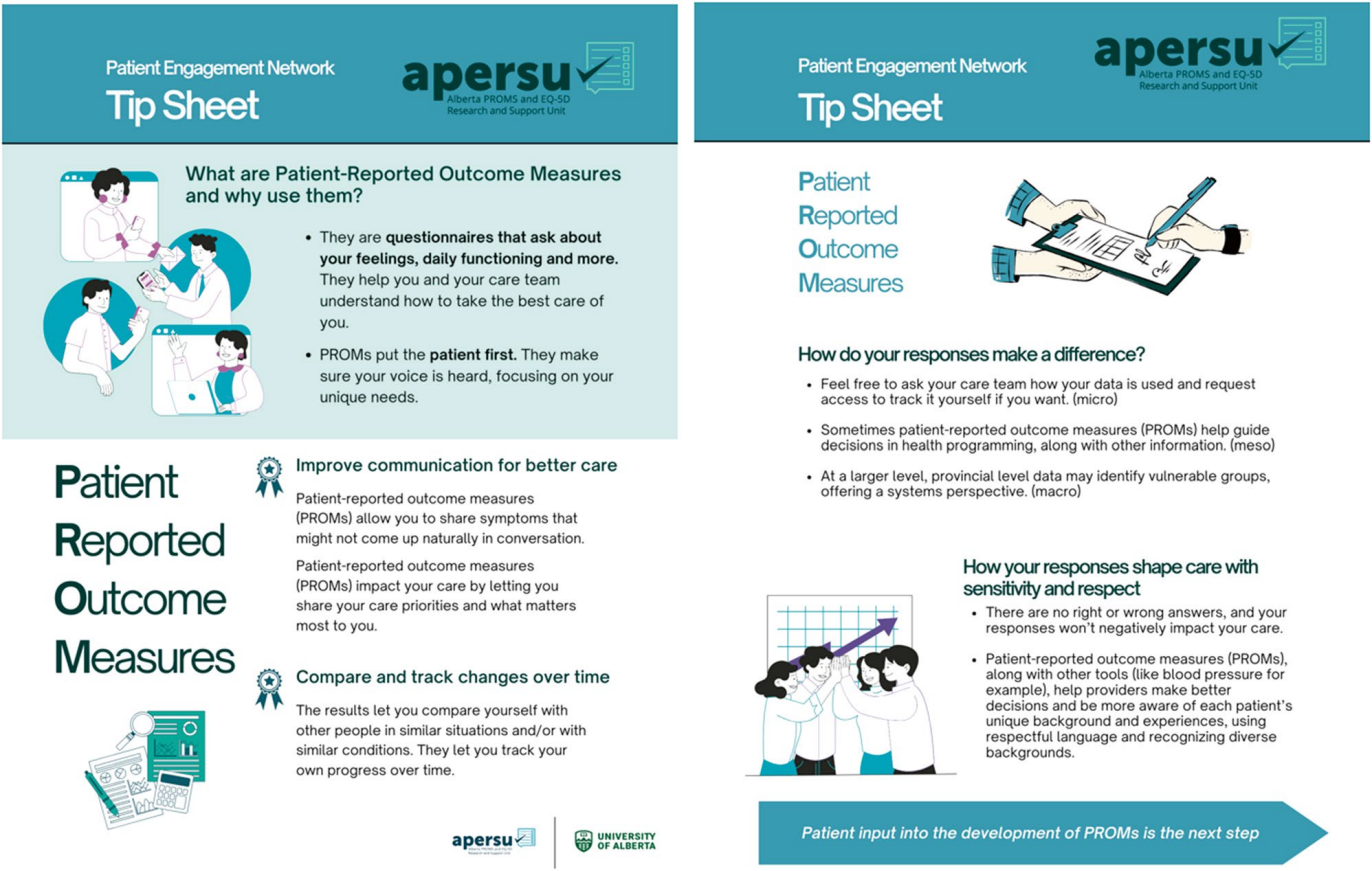
APERSU-PEN Tip Sheet

The co-development of a patient-friendly PROMs Tip Sheet (Fig. 2) has been a major milestone, addressing a gap in PROMs awareness amongst patient groups and networks. It communicates the value of PROMs patient-to-patient and addresses some common misconceptions previously raised by the network members. Its content was driven by experiences, opinions, stories and perceptions of PROMs. For example, one APERSU-PEN member wondered where the data was stored, if anyone even reviewed it and how it might impact the care received. The Tip Sheet addressed this by stating “There are no right or wrong answers, and your responses won’t negatively impact your care”.

Over several months, the Tip Sheet was co-designed, reviewed and finalized to ensure clarity, accessibility and relevance. The final resource, as illustrated in Fig. 2, is now available on APERSUs website (https://sites.google.com/ualberta.ca/apersu/about-us/patient-engagement-network), serves as a foundational tool for patient education and engagement. Future efforts will focus on broadening accessibility, including translations and alternative formats.

Beyond resource development, the APERSU-PEN has actively contributed to knowledge mobilization, sharing insights at various meetings and conferences, as further shown and explained in Table 1. Members attend quarterly meetings (at a minimum) to engage in capacity building activities, knowledge sharing and strategic planning. These efforts show the high level of engagement and motivation with the APERSU-PEN, with network members not only advocating for PROMs in research and routine care, but spreading awareness in their own networks. Rather than being passive stakeholders, members of the APERSU-PEN have co-led the selection of tools, shaped priorities, co-created knowledge translation strategies and guided outreach efforts within APERSU and other research studies and committee work. This model has strengthened APERSU’s ability to embed and sustain meaningful patient engagement into its work. Furthermore, these efforts reflect a high level of engagement consistent with the ‘collaborate’ and ‘empower’ categories of the IAP2 spectrum [7], where patients are actively involved in decision-making and co-leadership.

## Challenges and overcoming them

As noted above, an initial challenge in PPIE was the broad scope of work for APERSU, where there is no focus on a particular patient population, such as defined by a condition (e.g., diabetes, kidney disease), by age (e.g., elderly) or sex (e.g., women’s health). Rather, the remit of APERSU’s work was the whole healthcare system, supporting the implementation of PROMs in whatever clinical setting was interested in pursuing. Because of this, we approached the creation of APERSU-PEN as a “*network of networks and organizations*” (Fig. 1) rather than just a sample of patients with a particular condition. Indeed, the well-trained APERSU-PEN members are acutely aware that each of them represents one patient perspective and are conscientious of others when providing their views and feedback. We believe this approach has been one of the most important successes in the creation of our APERSU-PEN.

One of the biggest challenges in establishing APERSU-PEN was considering compensation processes and structures as fair compensation is best practice for meaningful engagement [12, 14]. Dedicated funding to support the members, network activities and outputs was secured as part of APERSU’s operational funding from the provincial health organizations. This funding was intentionally built into the proposal through discussions with the APERSU Board of Directors, supported by evidence of the networks’ value and impact. Maintaining a specific budget line for PPIE has been essential to sustaining relationships and ensuring the ongoing viability of the network. In practice, compensation is provided through institutional mechanisms and AbSPORU-supported guidelines. For example, issuing individual gift cards to acknowledge participation in meetings, material review and other network activities. Additional expenses, such as conference registration, travel and accommodations are reimbursed in accordance with institutional policies for non-employees, reducing barriers to participation and supporting equitable involvement. Another challenge in engaging with already busy patient partners is the juggling of multiple projects, health-related matters and priorities. Flexible meeting options were offered, with a variety of options for times and days (evenings and weekends) using a scheduler for everyone to indicate availability preferences. All meetings are held virtually through a mutually agreed upon accessible platform, recorded and shared internally for any members unable to attend. Minutes and notes are circulated within a week to all members regardless of attendance. To date, all meetings have had over 80% participation from members. Any co-development activities were voluntary based on interest and capacity, and many activities were directed by the needs, interests and availability of the group. This ensured those who wanted to participate, could, on their own schedule, including across time zones and participating remotely when necessary. Document sharing practices are facilitated using cloud-based servers, allowing all network members to access documents, educational materials and activities at any time, from anywhere.

Despite these challenges, and the time taken to address them, the overall value-added by the APERSU-PEN is significant. The network continues to increase awareness and knowledge of PROMs within patient communities, creating more opportunities for meaningful engagement with patients. It also has laid the groundwork for future collaborations on specific projects, to consider PROMs when discussing outcomes, and expanded opportunities to incorporate PROMs into other initiatives, guided by the priorities and direction of patients.

## The value of the APERSU-PEN from all perspectives

The APERSU-PEN brings together patients and researchers in a way that transforms both the culture and practice of healthcare. For example, researchers hearing a patient refer to themselves as the true “end-user” of PROMs is a powerful reminder of why this work matters. It’s not just about the data, it’s about shifting systems and mindsets to reflect what patients value and need, and involving them at multiple stages of PROMs implementation. As one APERSU collaborator noted, this innovation is “as much about a shift in culture as it is about a shift in practice and policy”, embedding patient-reported data into everyday care. Another spoke of a key learning: “Patients don’t mind seeing and hearing the word *patients*, over and over again. We don’t always have to refer to PROMs using the acronym.” From the patient perspective, being part of the process means having a voice in what gets measured and why. As one APERSU-PEN member shared, “I can be proud of you guys [researchers] for thinking of patients as the center,” while another emphasized that system measures alone are not enough, that patient-reported outcomes are crucial to understanding the patients’ perspective. This mutual learning and respect exemplifies the value of the APERSU-PEN to PROMs research, routine care, health organizations and the healthcare system.

## Future directions, implications and next steps

Looking ahead, evaluation of the APERSU-PEN model will be an important next step to understand its impact on PROMs use and patient-centered care. We plan to assess outcomes through both process and impact measures, including member satisfaction, perceived influence on PROMs-related activities, and evidence of increased PROMs uptake within research and clinical settings. First, it is important to evaluate the experiences of APERSU-PEN members to understand what currently works well within the network, and what can be improved. Evaluation of the patient engagement process presents an opportunity for members to reflect on their experiences and recommendations for the network. One validated evaluation survey we are considering is the Patient Engagement in Research Scale (PEIRS −22) [15]. The aim is to periodically survey members and include open-ended questions. Second, we have scheduled annual individual “check-in” meetings with members to assess how their contributions have shaped PROMs selection, interpretation or implementation decisions within their work. Third, APERSU tracks the number of research projects, clinical programs and Primary Care Networks have adopted PROMs, and can compare these numbers over time alongside APERSU-PEN activities.

Another consideration for the future membership of APERSU-PEN is to increase diversity among our group, extending our network beyond health organizations to other community groups with a vested interest in health and navigating healthcare. We used LinkedIn and email to schedule four meet-and-greet meetings with other community organizations (e.g., Imagine Citizens, Alberta International Medical Graduates Association) to promote our network, as well as linked local research projects to specific APERSU-PEN members based on interest and priorities.

Engagement of patients and families in the development, implementation, education, and dissemination of PROMs is vital for their uptake and ongoing use. Patients can be involved as partners at all stages of PROMs research and implementation. Few studies have engaged patients as partners in setting priorities for what outcomes to measure, advising on tools, development, education, and implementation practices [9, 16–18]. As well, we recognize this model may present challenges in other health systems and cultures. In contexts where PPIE is more institutionally and clinically driven, additional efforts may be needed to build trust, establish shared purpose and adapt engagement strategies to local structures and norms. However, we can say it’s worth the time, effort and money to involve patients at any and all stages, and there are plenty of guides and resources available to help get started.

## Data Availability

Not applicable.

## Abbreviations

AbSPORU: Alberta Strategy for Patient-Oriented Research Support Unit
APERSU: Alberta PROMs and EQ-5D Research and Support Unit
APERSU-PEN: Alberta PROMs and EQ-5D Research and Support Unit Patient Engagement Network
IAP2: International Association of Public Participation
NIHR: National Institute for Health and Care Research
PaCER: Patient and Community Engagement Research
PIERs: Patient Engagement in Research Scale
PPIE: Patient and Public Involvement
PROMs: Patient-Reported Outcome Measures
PWLE: Person With Lived Eexperience
SPOR: Strategy for Patient-Oriented Research
